# How Not to Recognize Disease

**Published:** 1907-12

**Authors:** 


### How Not to Recognize Disease.

That some physicians diagnose ailments, in certain cases, not from actual examination of the patient, but from what is told them by other persons, is the somewhat disquieting accusation made by the *Dietetic and Hygienic Gazette* (October). Says a writer in this magazine:

"We have frequently asked ourselves how many times a diagnosis is made by physicians, especially in somewhat obscure cases, by suggestion from the family or friends. Let us illustrate by citing a case, which for our purposes may be considered a hypothetical one. A lady is taken suddenly dangerously ill at midnight, the family physician is hurriedly sent for, and is told by the husband and mother before entering the sick chamber that the patient partook of a portion of a bad fish for dinner. The mother is sure the fish was bad because it tasted tainted, further stating that she did not eat of her portion, rejecting the first bite; therefore, the patient must be suffering from ptomain poisoning. The physician finds his patient suffering greatly; there are nausea and vomiting, some fever, with rapid and feeble pulse, cramps in the limbs, and tenderness of the abdomen. Calomel is given, followed by a saline cathartic, brandy, and an opiate, with hot cloths over the abdomen. Thus the patient is left until noon the next day, with instructions to the attendant to give opiates often enough to quiet pain. Thirty-six hours from the onest of attack patient dies. Autopsy requested and made, disclosing death from a ruptured appendix. Now the question arises, would a proper diagnosis have been made if the suggestion of ptomain poisoning had not been given, and in that case could the patient have been saved by timely surgical procedures? The moral of this illustration is for the medical attendant to make the diagnosis in the sick chamber after examination of the patient, and not in an anteroom."—*Literary Digest.*

